# Non-Coding RNAs in Regulating Fat Deposition in Farm Animals

**DOI:** 10.3390/ani15060797

**Published:** 2025-03-11

**Authors:** Jingxuan Li, Xueyan Zhao, Yanping Wang, Jiying Wang

**Affiliations:** 1 Shandong Key Laboratory of Animal Disease Control and Breeding, Institute of Animal Science and Veterinary Medicine, Shandong Academy of Agricultural Sciences, Jinan 250100, China; 15927409635@163.com (J.L.); zhaoxueyan0102@163.com (X.Z.); wangyanping03@163.com (Y.W.); 2 Key Laboratory of Livestock and Poultry Multi-Omics of MARA, Jinan 250100, China

**Keywords:** adipogenesis, miRNA, lncRNA, circRNA, farm animals

## Abstract

Fat deposition represents a crucial feature in the expenditure of physical energy and affects the meat quality of farm animals. It is regulated by multiple genes and regulators. Of them, non-coding RNAs (ncRNAs) play a critical role in modulating the fat deposition process. As well as being an important protein source, farm animals can be used as medical models, so many researchers worldwide have explored their mechanism of fat deposition. This article summarizes the transcription factors, regulatory genes, and signaling pathways involved in the molecular regulation process of fat deposition; outlines the progress of researching the roles of microRNAs (miRNAs), long ncRNAs (lncRNAs), and circular RNAs (circRNAs) in fat deposition in common farm animals including pigs, cattle, sheep, ducks, and chickens; and identifies scientific problems in the field that must be further investigated. It has been demonstrated that ncRNAs play a critical role in regulating the fat deposition process and have great potential in improving meat quality traits.

## Simple Summary

Fat deposition is a complex biological process regulated by many genes and regulators. Recently, more and more studies have demonstrated that ncRNAs serve as regulators in gene expression networks and participate in various biological processes, including in fat deposition in farm animals. This review systematically summarizes research progress on the roles of non-coding RNAs (ncRNAs) in regulating fat deposition across extensively researched livestock species and identifies several key scientific challenges in this emerging field. This review provides a theoretical foundation for future in-depth research on the regulatory mechanisms of fat deposition in farm animals and the genetic improvement of meat quality.

## 1. Introduction

Adipose tissue is loose connective tissue found under the skin and surrounding organs and tissues. It contains mature and preadipocytes; nerves; and mesenchymal matrix/stem, vascular endothelial, contractile, and a range of immune cells [1,2]. Fat deposition in adipose tissue is the process of storing excess energy, which can be released at appropriate times to maintain the balance of metabolism. Farm animals are the most important protein source and are used as medical models. Their fat deposition influences many economically important traits, such as backfat thickness, subcutaneous fat (SCF) and intramuscular fat (IMF) content, and lean meat percentage [3]. Therefore, understanding the regulatory network of fat deposition could improve farm animal meat production and quality.

Fat deposition is a complex biological process. Adipocytes are generated by mesenchymal stromal cells (MSCs) which undergo adipogenesis through specific signaling molecules and develop into preadipocytes. Then, mitotic clonal expansion and terminal differentiation occur to form mature adipocytes under the control of key transcription factors [4,5,6]. This process begins with the arousal of a member of the activating protein-1 (AP-1) transcription factor family and subsequently inducing the expression of peroxisome proliferator-activated receptor gamma (PPAR-γ), a key pro-fat transcription factor. Next, the transcription factors of signal transducers and activators of transcription (STATs)—members of the Krüppel-like factor (KLF) protein, sterol response element-binding protein-1 (SREBP-1), and CCAAT enhancer binding protein families (C/EBPs)—promote adipocyte maturation. Preadipocytokin-1 (Pref-1) and members of the GATA and Wnt families, as negative inhibitors of adipocyte differentiation, also coordinate this process [7].

Thus far, many researchers have conducted extensive research on the mechanism of fat deposition. In recent years, a growing number of studies have demonstrated that non-coding RNAs (ncRNAs), including microRNAs (miRNAs), long non-coding RNAs (lncRNAs), and circular RNAs (circRNAs), are extensively involved in various adipogenesis processes [8,9]. As an emerging player in regulating fat deposition, there is an increasing amount of research on the ncRNA-led regulation of fat deposition in farm animals, especially in pigs, cattle, sheep, ducks, and chickens. Here, the current literature on ncRNA identification and functional characterization in animal adipogenesis is reviewed, and a comprehensive synthesis of the published findings that delineate the regulatory mechanisms mediated by these ncRNAs is presented. This review aims to elucidate the emerging roles of ncRNAs as epigenetic modulators in the fat deposition processes.

## 2. Molecular Regulation of Fat Deposition

Fat deposition is a complex process regulated by many key transcription factors such as PPARγ, C/EBPα, KLF5, etc. [8,10]. These factors are generally regarded as marker genes in the fat deposition process. Meanwhile, fat deposition is also regulated by numerous signaling pathways, including Janus kinase 2 (JAK2)-signal transducer and the activator of transcription 3 (STAT3), cyclic AMP (cAMP), Wingless/Integrated (Wnt), etc. [11,12,13]. Many regulatory genes can directly target key transcription factors or indirectly activate adipocyte-specific genes through these signaling pathways to regulate fat deposition.

Among the transcription factors, the key ones are PPAR-γ and the C/EBPs. PPAR-γ, a member of the nuclear receptor superfamily, is necessary for fat deposition. The majority of the repressors and activators of fat deposition modulate PPAR-γ expression and activity. Some studies using in vitro models have consistently shown that PPAR-γ mRNA is induced by several transcription factors, including C/EBP-β, C/EBP-δ, early B-cell factor 1, and KLF5. The repressors of adipogenesis, such as GATA2, KLF2, and C/EBP-homologous protein (CHOP), attenuate PPAR-γ expression. Embryonic fibroblasts obtained from KLF5^+/−^ mice undergo attenuated adipocyte differentiation. KLF5 expression is induced by C/EBP-β and C/EBP-δ. KLF5, in turn, acts in concert with C/EBP-β/δ to activate the PPAR-γ2 (a protein subtype of PPAR-γ) promoter [14]. Unlike KLF5, the ectopic expression of KLF2 in 3T3-L1 preadipocytes significantly disrupts lipid accumulation by repressing PPAR-γ2 gene expression [15].

C/EBP family members are the master regulators of adipocyte terminal differentiation, including C/EBP-α, C/EBP-β, C/EBP-γ, C/EBP-δ, CHOP, and so on. C/EBP-β and C/EBP-δ are expressed in the early stages of adipocyte differentiation and induce the transcription of C/EBP-α and PPAR-γ to initiate adipocyte differentiation [16,17,18]. C/EBP-α can directly induce the expression of many adipogenic genes. Wu et al. also showed that C/EBP-α-deficient adipocytes form fewer lipid droplets and are unable to induce the expression of endogenous PPAR-γ, which indicates that C/EBP-α plays a significant role in fat deposition. The cross-regulation between PPAR-γ and C/EBP-α is the key component of the regulation of adipocyte differentiation [19].

Similarly, many pathways are involved in the process of fat deposition, such as JAK2 STAT3, cAMP, and Wnt [12,20,21,22]. The JAK2-STAT3 pathway regulates the pre-differentiation of adipocytes by activating the transcription of C/EBP-β. Some studies have shown that the signal transducers and activators of transcription (STATs) play a role in activating adipogenic differentiation mainly by promoting the expression of the downstream gene PPAR-γ [13]. In addition, STAT3 stimulates the proliferation of adipogenic stem cells at the pre-differentiation stage, thereby promoting fat deposition [23]. The cAMP signaling pathway regulates fat deposition mainly via mediation through the cAMP-responsive element-binding protein (CREB). Fox et al. showed that cAMP stimulates the phosphorylation and binding of CREB to the promoter of gene cyclin D1, thereby allowing for adipogenesis to proceed [24]. The Wnt signaling pathway is a widely studied negative regulator of fat deposition. As we know, the expression levels of PPAR-γ, C/EBP-α, and adductive 1 (ADD1), as well as of fatty acid-binding protein (FABP) 4 and adiponectin (APM1), increase when 3T3-L1 cells induce adipogenic differentiation. However, PPAR-γ, C/EBP-α, ADD1, FABP4, and APM1 are almost absent in models in which the Wnt signaling pathway is activated [25].

**Figure 1:**
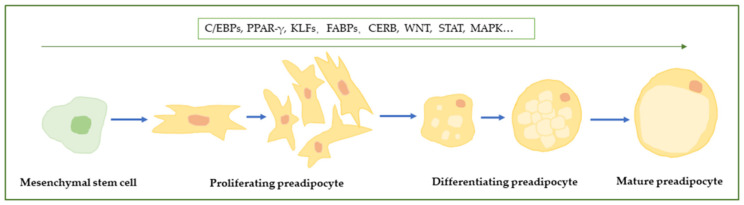
The process of fat deposition. Mesenchymal stem cells (found in the vascular stroma of adipose tissue) are multidirectional and are capable of transforming into preadipocytes. The preadipocytes, under adequate adipogenic stimuli, undergo mitotic clonal expansion and differentiation to turn into mature adipocytes.

In addition to the pathways mentioned above, insulin-like growth factor 1 (IGF-1), glucocorticoid (GC), mitogen-activated protein kinases (MAPKs), hedgehog (Hh), and rat sarcoma (RAS) also play essential roles in the process of fat deposition [26,27,28,29,30,31,32,33,34]. The process of fat deposition is illustrated in Figure 1.

## 3. Regulatory ncRNAs and Fat Deposition

In general, ncRNAs can be further classified according to different criteria. Usually, they are classified by function into housekeeping RNAs and regulatory RNAs. Housekeeping RNAs mainly include transfer RNAs, ribosomal RNAs, small nucleolar RNAs, and small nuclear RNAs. Regulatory ncRNAs are mainly further classified by length as lncRNAs (>200 nt) or small regulatory ncRNAs (18–200 nt), including miRNAs, piwi-associated RNAs, and endogenous siRNAs. Additionally, circRNAs span lengths of 20–1600 nt, thus partly overlapping with the length of short ncRNAs [35,36]. Among them, miRNAs, lncRNAs, and circRNAs have been extensively studied, and their roles in regulating fat deposition in farm animals is reviewed as follows.

### 3.1. miRNAs Regulating Fat Deposition in Farm Animals

miRNAs are a class of ncRNAs that are 18–25 nt in length and regulate gene expression by binding to the 3′ untranslated region (3′ UTR) of target genes, thereby inhibiting protein synthesis mainly by repressing translation or promoting mRNA decay [36]. miRNAs are the most well-studied ncRNAs due to their strongly conserved nature and the well-established research methods and databases used. In recent years, some researchers have identified miRNAs in animal adipose tissue using high-throughput sequencing technology, explored their functions through bioinformatic analysis and functional experiments, and demonstrated that miRNAs are one type of ncRNA regulating fat deposition in farm animals. The miRNAs that promote or inhibit IMF and SCF deposition in the most common livestock animals (cattle, sheep, pigs, and chickens) are reviewed below.

#### 3.1.1. miRNAs Positively Regulating Fat Deposition

In 2010, Chao et al. were the first to identify and clone miRNAs from porcine adipose tissue. Among these miRNAs, two families were found to be expressed at a high frequency: *miR-199* (21.3%) and *let-7* (14.4%) [37]. Subsequently, a series of miRNAs that promote fat deposition in pigs were also described. Li and colleagues identified *miR-103*, which reportedly promotes porcine preadipocyte differentiation and may act through the putative target gene retinoic acid induced 14 (*RAI14*) [38]. Tumor necrosis factor-alpha (TNF-α) is a key regulator that has been implicated in adipocyte metabolism, with effects that include the promotion of lipolysis and the potent inhibition of adipocyte differentiation [39,40]. Some studies have shown that *miR-181a* promotes porcine adipogenesis by targeting *TNF-α*, consequently altering the expression of the genes regulated by TNF-α [41]. Similarly, *miR-140-5p/b* and *miR-196a* have been identified as positive regulators of the differentiation of porcine preadipocytes, with further research showing that forkhead box transcription factor O1 (*FOXO1*) is a direct target of *miR-15a/b* [42,43].

In addition to pigs, in recent years, many miRNAs that promote fat deposition in cattle, goats, and chickens have also been identified. For example, *miRNA-143* reportedly promotes differentiation but inhibits the proliferation of bovine preadipocytes [44]. *miR-1271* and *miR-381* may promote the adipogenic differentiation of Yanbian cattle preadipocytes by targeting activating transcription factor 3 (*ATF*3) and potassium channel tetramerization domain containing 15 (*KCTD15*), respectively [45,46]. *miR-330* promotes the bovine adipogenesis of intramuscular preadipocytes by targeting Sestrin3 to activate the Akt-mTOR signaling pathway [47]. In goats, *miR-196a* has been reported to promote IMF deposition by targeting mitogen-activated protein kinase kinase kinase 1 (MAP3K1) and activating the phosphatidylinositol-3-kinase/protein kinase B (PI3K-Akt) pathway [48].

In chickens, *miR-122-5p* directly targets FABP5 to promote the differentiation of preadipocytes [49]. *miR-125b-5p* promotes preadipocyte differentiation, affecting adipogenesis in chickens’ abdominal adipose tissues, at least in part by downregulating acyl-CoA synthetase bubblegum family member 2 (*ACSBG2*) [50]. Similarly, *miR-15a* and *miR-140-5p* positively regulate the differentiation of chicken preadipocytes [51,52].

#### 3.1.2. miRNAs Negatively Regulating Fat Deposition

*miR-145* is upregulated during porcine differentiation, inhibiting adipogenesis through the targeting of insulin receptor substrate 1 (*IRS1*) [53]. *miR-146a-5p* inhibits TNF-α-induced adipogenesis via the targeting of the insulin receptor in primary porcine adipocytes [54]. Both *miR-29b* and *miR-29c* negatively regulate porcine adipogenesis by targeting C1q/tumor necrosis factor-related protein 6 (*CTRP6*) [55]. *miR-125a-5p* also inhibits the differentiation of porcine intramuscular preadipocytes by directly targeting Krüppel-like transcription factor 13 (*KLF13)* [56]. *miR-199a-5p* significantly promotes cell proliferation and attenuates lipid deposition in porcine adipocytes through the absorption of caveolin-1 (*CAV-1*) [57], whereas *miR-127* activates preadipocyte proliferation and suppresses differentiation through the targeting of homeobox C6 (*HOXC6*) [58]. *miR-218-5p* exerts an inhibitory effect on porcine preadipocyte differentiation by inhibiting acyl-CoA synthetase long-chain family member 1 (*ACSL1*) expression [59], while *miR-26a* also plays the same role by regulating acyl-CoA dehydrogenase medium chain (ACADM) and acyl-Co A synthetase 1 (ACSL1) [60]. Meanwhile, *miR-29c* inhibits the proliferation and adipogenic differentiation of porcine bone marrow stromal cells by targeting insulin-like growth factor 1 (*IGF1*) [61].

In cattle, the overexpression of *miR-130a/b* leads to significantly reduced lipid droplet formation during the fat deposition process and inhibits the expression of adipocyte differentiation-related genes. A further assay verified that *miR-130a/b* significantly affects *PPAR-γ* and *CYP2U1* expression by directly binding to their 3′ UTRs [62]. *miR-107* suppresses bovine adipocyte differentiation and lipogenesis by directly targeting apolipoprotein C2 (*APOC2*) [63]. *miR-484* inhibits adipogenic differentiation but does not altering the fatty acid composition of bovine intramuscular adipocytes by interacting with its target gene mitogen-activated protein kinase kinase kinase 9 (MAP3K9) [64]. *miR-10167-3p* also inhibits bovine adipocyte differentiation and promote bovine adipocyte proliferation by targeting T cell factor 7-like 1 gene (TCF7L1) [65]. Moreover, *miR-365-3p* significantly inhibits lipid accumulation and decreases the triglyceride content in bovine adipocytes by targeting FK506-binding protein 5 (FKBP5) [66].

In sheep, *miR-340-5p* reportedly acts as an inhibitor of intramuscular adipocyte differentiation through the targeting of activating transcription factor 7(*ATF7*) [67], whereas *miR-27a* and *miR-124-3p* inhibit the differentiation of preadipocytes by targeting the 3’ UTRs of retinoid X receptor alpha (*RXRα*) and *C/EBP-α*, respectively [68,69]. Luo et al. demonstrated a dual regulatory role for *miR-136* in adipogenesis in which *miR-136* both promotes preadipocyte proliferation by elevating the *IGF1* expression level and inhibits preadipocyte differentiation by repressing *PPAR-γ* and *C/EBP-α* expression [70]. Several other miRNAs, including *miR-432*, *miR-369-3p*, and *miR-33a*, also inhibit fat deposition [71,72,73].

Using chickens, Sun et al. discovered that *gga-miR-18b-3* inhibits intramuscular adipocyte differentiation through the targeting of acyl-CoA thioesterase 13 (*ACOT13*) [74]. *miR-106-5p* was found to inhibit the proliferation and adipogenic differentiation of chicken abdominal preadipocytes by directly targeting Krüppel factor 5 *(KLF15)* [75]. In addition, *miR-128-3p* and *miR-223* reportedly inhibit chicken intramuscular adipocyte differentiation by downregulating farnesyl diphosphate synthase (*FDPS*) and glycerol-3-phosphate acyltransferase (*GPAM*), respectively [76,77]. The miRNAs mentioned above that regulate fat deposition in farm animals are summarized in Table 1.

### 3.2. lncRNAs Regulating Fat Deposition in Farm Animals

lncRNAs are a group of RNAs in eukaryotes with a length of more than 200 nt that are mainly transcribed by RNA polymerase II [78]. They were initially misclassified as non-coding genes and were considered to be nonfunctional byproducts of transcription [8,79]. However, some lncRNAs encode small peptides [80,81]. Many lncRNAs have been identified in animals over the last decade, with some playing important roles in regulating fat deposition. Currently, research on the lncRNA-mediated regulation of animal fat deposition mainly utilizes sequencing to screen for specific lncRNAs in various sites of adipose tissue, or it uses functional and mechanistic techniques. The lncRNAs involved in the fat deposition process in farm animals are reviewed as follows.

#### 3.2.1. Screening of lncRNAs in Adipose Tissue

Using RNA-seq and other technologies, a large number of lncRNAs, many of which are involved in fat deposition, have been detected in the genomes of farm animals. Huang et al. comparatively analyzed the gene expression profiles of IMF and SAT in Laiwu and Large White pigs, revealing differentially expressed (DE) lncRNAs that target the mRNAs involved in the PPAR and MAPK signaling pathways to play important roles in fat accumulation and adipogenic differentiation [82,83]. Similarly, Zhang et al. performed a comparative transcriptome analysis of the longissimus dorsi (LD) muscles of fat-type Laiwu pigs and commercial lean-type Duroc × Landrace × Yorkshire pigs. In their study, they identified 12 miRNAs and revealed two network modules in the LD muscle [84]. Furthermore, Li et al. comparatively analyzed the expression profiles of LD muscles in individual Laiwu pigs with extremely high and low IMF content, identified 180 DE lncRNAs, and observed that three genes involved in fat deposition, namely *SCD*, phosphoenolpyruvate carboxykinase 1 (*PCK1*), and adiponectin (*ADIPOQ*), were the targets of some of the DE lncRNAs [85].

Adipose tissue is deposited in different parts of sheep bodies, including the subcutaneous layer under the skin, around the viscera, and within the abdominal cavity. This is the same for pigs and cattle. Therefore, some research on adipogenesis in sheep has been carried out using these tissues. For example, Bao et al. analyzed the function and regulatory network of lncRNAs in lipid metabolism during muscle growth and development at four growth stages in Tibetan sheep. They identified 360 DE lncRNAs, some which transregulate target genes and further regulate the growth and development of muscle and intramuscular fatty acid metabolism [86]. Liu et al. investigated lncRNAs in the SAT of Duolang and small-tailed Han sheep and identified 107 DE lncRNAs, of which *LOC105616076*, *LOC114118103*, *LOC105607837*, *LOC101116622, LOC105603235*, and others play key regulatory roles in the biosynthesis of unsaturated fatty acids through the regulation of target genes [87]. Research on the lncRNAs involved in sheep IMF deposition has also been conducted. Han et al. used RNA-seq to identify sixty-one DE lncRNAs during intramuscular lipid deposition in Aohan fine-wool sheep, obtaining seven candidate lncRNAs that may regulate lipid deposition and constructing an lncRNA–mRNA co-expression network [88]. The tail is where fat is deposited in sheep [89]. Recently, many studies on the lncRNA-mediated regulation of sheep tail fat deposition have been carried out. Ma et al. compared the transcriptomic differences among fat-tailed Lanzhou, small-tailed Han, and Tibetan sheep, identifying 68 DE lncRNAs, of which *TCONS_00372767*, *TCONS_00171926*, *TCONS_00054953*, and *TCONS_00373007* showed potential associations with tail adipose tissue enlargement [90]. Bakhtiarizadeh et al. also performed a comparative transcriptome analysis of fat-tailed (Lori–Bakhtiari) and thin-tailed (Zel) Iranian sheep breeds, identifying seven DE lncRNAs with potential involvement in sheep tail fat development [91]. To further understand the regulatory mechanisms underlying tail fat deposition at different growth stages, He et al. conducted targeted transcriptomic analyses of mRNAs and lncRNAs in the tail fat of Sunite sheep at 6, 18, and 30 months of age, discovering 148 DE lncRNAs that may participate in the regulation of its growth and development [89].

Furthermore, a study by Li et al. in 2016 investigating lncRNAs in bovine preadipocytes and differentiated adipocytes identified 16 DE lncRNAs, which provided the first comprehensive annotation of lncRNAs in bovine adipogenesis [92]. Yang et al. performed transcriptome sequencing of the abdominal adipose tissue of ducks with high and low abdominal fat percentages, and they identified 633 DE mRNAs and 214 DE lncRNAs. The target genes of the DE lncRNAs were enriched in pathways associated with fat synthesis and metabolism, indicating that the lncRNAs play important roles in abdominal fat deposition [93]. Zhang et al. conducted the first analysis of lncRNA and mRNA expression during the differentiation of abdominal preadipocytes in chickens, identifying 27,023 novel lncRNAs, including several (e.g., *XLOC_068731* and *XLOC_022661*) that may play potential key roles in preadipocyte differentiation [94]. Subsequently, Chen et al. systematically analyzed the DE lncRNAs, miRNAs, and mRNAs in differentiated and undifferentiated preadipocytes, constructing a competing endogenous RNA (ceRNA) regulatory network and identifying eight crucial ceRNA interactions related to chicken preadipocyte differentiation [95].

#### 3.2.2. lncRNAs Positively Regulating Fat Deposition

Certain lncRNAs positively regulating fat deposition have been studied in pigs. For example, *IMFlnc1* may reduce the *miR-199a-5p*-mediated suppression of CAV-1, thereby promoting fat deposition [96]. *lncMYOZ2* was shown to facilitate the adipogenesis of porcine preadipocytes in a denosylhomocysteinase (AHCY)/DNMT1-dependent manner, balancing the expression of myozenin 2 (MYZO2) [97], whereas *lncIMF2* was found to promote adipogenesis in porcine intramuscular preadipocytes through the sponging of *miR-217* [98]. Several antisense lncRNAs (AS lncRNAs) involved in the regulation of porcine fat deposition have been described. For example, *PU.1*, the first reported porcine AS lncRNA to be implicated in adipogenesis, is transcribed from the porcine gene *PU.1*. It was found that its expression level is relatively high only in spleen and adipose tissues, and it first increases and then decreases during porcine adipocyte differentiation. Functionally, its knockdown in porcine primary preadipocytes has been shown to cause the downregulation of PPAR-γ and FASN along with the inhibition of lipid droplet formation. Mechanistically, it forms a sense–antisense RNA duplex with *PU.1* mRNA to inhibit its translation and promote fat deposition [99].

In cattle, the lncRNAs promoting fat deposition primarily include the following. It was found that *lncFAM200B* promotes the proliferation of bovine preadipocytes, and its two single-nucleotide polymorphisms (SNPs) are associated with body measurement traits in Nanyang cattle [100]. Further studies by Zhang et al. demonstrated that the target gene of *lncFAM200B*, *bta-miR-6529a*, plays a negative role in regulating the proliferation and differentiation of yak preadipocytes [101]. *BIANCR* enhances adipogenesis by inhibiting the activation of the extracellular signal-related kinase 1 and 2 (ERK1/2) signaling pathway [102], and *NDUFC2-AS* promotes adipogenic differentiation by upregulating the expression levels of thyroid hormone responsive protein (*THRSP*) and *C/EBP-α* in buffalo [103]. Meanwhile, *SERPINE1AS2* plays a crucial role in the adipogenesis of bovine intramuscular adipocytes by modulating the expression of plasminogen activator inhibitor-1 (*PAI1*) [104].

There have also been several reports on lncRNAs regulating fat deposition in chickens and ducks. The first study showing lncRNAs playing a functional role in chicken adipogenesis was reported in 2019. In the study, the results showed that *IMFNCR* acts as a ceRNA to sequester *miR-128-3p* and *miR-27b-3p*, leading to increased PPAR-γ expression levels and promoting intramuscular adipocyte differentiation [105]. *lncAD* promotes the adipogenic differentiation of intramuscular preadipocytes by cis-regulating the expression of the upstream gene thioredoxin reductase 1 (TXNRD1) in chickens [106]. *ZFP36L2-AS* promotes IMF deposition by positively regulating the expression of the key genes involved in fatty acid synthesis. Meanwhile, in vivo, *ZFP36L2-AS* facilitates IMF deposition in chicks [107]. *LNC6302* promotes chicken abdominal preadipocyte differentiation by activating carnitine transporter 2 in a cis-regulating manner [108]. In 2016, a study by Li et al. provided the first comprehensive annotation of lncRNAs in bovine adipogenesis [92]. Using Ribo-Zero RNA-seq, they investigated lncRNAs in bovine preadipocytes and differentiated adipocytes, identifying 16 DE lncRNAs.

#### 3.2.3. lncRNAs Negatively Regulating Fat Deposition

Compared with lncRNAs that positively regulate fat deposition in farm animals, relatively few lncRNAs that negatively regulate fat deposition in farm animals have been reported to date. In pigs, *lncIMF4* was found to be associated with fat deposition in the RNA-seq analysis of intramuscular adipocytes from fat-type Bamei pigs and lean-type Yorkshire pigs. The knockdown of *lncIMF4* facilitates IMF deposition through the attenuation of autophagy to repress lipolysis [109]. Shi et al. found that *LOC106505926* inhibits the differentiation of porcine preadipocytes by directly binding to fatty acid synthase (FASN) [110].

**Figure 2:**
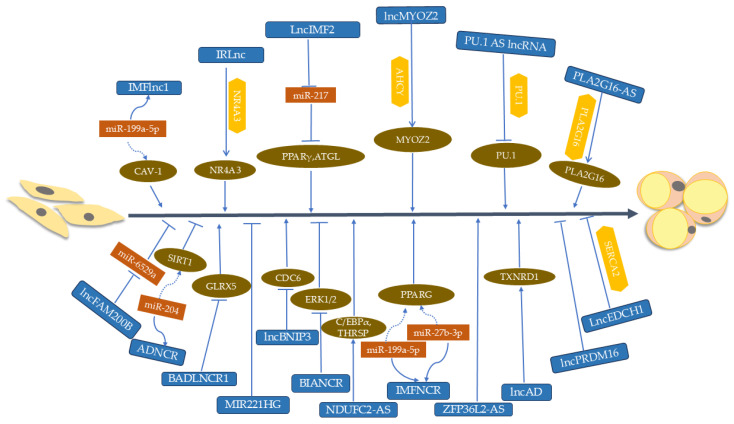
The regulatory mechanisms of lncRNAs (shown as blue boxes) involved in fat deposition in farm animals. The orange boxes, yellow hexagons, and brown ovals represent miRNAs, binding proteins, and target genes, respectively. The dotted line and the solid line indicate the regulation between lncRNAs and their downstream genes.

In cattle, *ADNCR* inhibits adipocyte differentiation by functioning as a ceRNA for *miR-204* [92]. Another lncRNA, *BADLNCR1*, negatively regulates adipocyte differentiation by significantly inhibiting C/EBP-α enhancement at the glutaredoxin 5 (*GLRX5*) promoter located immediately downstream [111]. Multiple genes can be transcribed from a single genomic sequence. For example, the lncRNA *MIR221HG* located on the bovine X chromosome overlaps with *miR-221* in the genome. Some researchers discovered that the inhibition of *MIR221HG* significantly intensifies adipocyte differentiation, as indicated by dramatic increases in the number of mature adipocytes and the expression levels of the adipogenic markers *PPAR-γ*, *C/EBP-α*, and *FABP4* [112]. The regulation of ubiquitination by lncRNAs might also play a role in the differentiation and proliferation of preadipocytes. *LncBNIP3*, a DE lncRNA between the IMF of Qinchuan cattle and that of Japanese Black cattle, was found to inhibit the proliferation of bovine intramuscular preadipocytes by directly regulating the expression of cell division control protein 6 (*CDC6*) [113]. In addition, *lncEDCH1* inhibits IMF deposition by binding with sarcoplasmic/endoplasmic reticulum calcium ATPase 2 (SERCA2) protein, and in vivo, it also impedes IMF deposition in chickens [114]. Figure 2 illustrates the most well-studied lncRNAs and their mechanisms for the regulation of fat deposition in farm animals. Additionally, the above-mentioned lncRNAs are summarized in Table 2.

### 3.3. circRNAs Regulating Fat Deposition in Farm Animals

circRNAs are primarily generated through the back-splicing of precursor mRNAs, and they lack 5’ caps and 3’ tails. They are single-stranded RNAs that form covalently closed loops. Therefore, circRNAs are not easily degraded by RNase R and are more stable than linear transcripts. Some circRNA molecules contain miRNA binding sites and can be used as molecular sponges of miRNAs [115]. With the recent development of high-throughput sequencing technology and circRNA-specific bioinformatics, a large number of circRNAs have been identified, some of which are reportedly related to fat deposition in farm animals. The circRNAs involved in the fat deposition process in farm animals are reviewed as follows.

#### 3.3.1. Screening of DE circRNAs in Adipose Tissue

Zhao et al. analyzed the circRNA expression profiles of the LD muscle of 2- and 12-month-old Aohan fine-wool sheep, identifying one hundred and four DE circRNAs and constructing a network diagram of circRNA–miRNA interactions that might be involved in the IMF deposition process. Using a dual-luciferase reporter assay, they were able to verify the targeting relationship of *circRNA4557–miR-149-5p* [116]. Huang et al. characterized the circRNA expression profiles of adipose tissues from young and adult Chinese buffalos, identifying two hundred and fifty-two DE circRNAs, two of which show a strong correlation with PRDM16 and may be regulators of buffalo fat deposition [117].

A number of circRNA sequencing and screening studies have been performed on pigs. For instance, Yousuf et al. performed a high-throughput whole-genome transcriptome analysis of IMF tissues from the LD muscles of Large White and Laiwu pigs, identifying two hundred and eighty-three DE circRNAs that include two key circRNAs (*circRNA-23437* and *circRNA-08840*) with potential binding sites for multiple miRNAs, regulating the whole network [118]. Li et al. detected eight hundred and eighty-three candidate circRNAs, among which twenty-six and twelve circRNAs were found to be DE in Landrace and Songliao pigs spread among the high- vs. low-backfat-thickness groups of each breed, respectively. Notably, they identified two vital circRNA regulators with the majority of the target genes enriched in biological functions related to adipogenesis in pigs [119]. Similar work has been carried out in our laboratory. In our study, the expression profiles of LD muscles between three Laiwu pigs with high IMF content and three with low IMF content were analyzed. A total of one hundred and five DE circRNAs were identified, and a basic source gene–circRNA–miRNA connective network was constructed using miRNA–circRNA binding site analysis, providing the basis for future studies of candidate circRNAs [85].

#### 3.3.2. circRNAs Positively Regulating Fat Deposition

In goats, *chi-circ_0006511* reportedly upregulates adipogenic differentiation determinants and further promotes intramuscular preadipocyte differentiation by sponging novel-miR-87, thereby regulating the expression of cluster of differentiation 36 (*CD36*) [120]. *circBDP1*, which is derived from the bovine B double prime 1 gene (*BDP1*), also plays a positive role in the proliferation and differentiation of bovine preadipocytes by competitively adsorbing *miR-181b* and *miR-204*, thereby affecting the expression of sirtuin 1 (*SIRT1*) and the trafficking regulator of GLUT4 1 (*TRARG1*) [121]. *circPPAR-γ* facilitates adipocyte differentiation and inhibits proliferation through the binding of *miR-92a-3p* and Yinyang 1 (*YY1*) in bovine primary adipocytes [122], while *circFLT1* exerts the same function by sponging *miR-93* in Qinchuan cattle [123]. *circMARK3* promotes the adipogenic differentiation of buffalo adipocytes and 3T3-L1 cells by upregulating the expression levels of the adipogenic marker genes *PPAR-γ*, *C/EBP-α*, and *FABP4* [124]. The newly identified bovine *circRNF111* functions as an *miR-27a-3p* sponge to rescue the inhibitory effect of *miR-27a-3p* on *PPAR-γ*, thereby promoting fat deposition [125].

In pigs, *circSETBP1* inhibits the proliferation and promotes the differentiation of porcine intramuscular preadipocytes through the *miR-149-5p*/CREB-regulated transcriptional coactivator (*CRTC*) axis [126]. Like *circSETBP1*, *circPPARA* promotes the differentiation of porcine intramuscular preadipocytes and hinders their proliferation by adsorbing *miR-429* and *miR-200b* to promote IMF deposition in pigs [127].

Wang et al. also explored the circRNAs that affect fat deposition in ducks, identifying one hundred and forty-one DE circRNAs, including *circ-PLXNA1*, that exhibit ceRNA networking. Further experiments showed that the number of lipid droplets, the adipogenic ability, and the triglyceride content of the preadipocytes decrease after the transfection of a small interfering RNA against *circ-PLXNA1*, indicating that *circ-PLXNA1* may act as a promoter of duck preadipocyte differentiation [128].

#### 3.3.3. circRNAs Negatively Regulating Fat Deposition

In cattle, *circINSR*, a circRNA with an inhibitory effect on fat deposition, was shown to inhibit preadipocyte adipogenesis by alleviating the inhibitory effect of *miR-15/16* on the target genes *FOXO1* and ethanolamine phosphotransferase (*EPT1*) [129]. Unlike its bovine counterpart, *circINSR* in sheep was found to inhibit the adipogenic differentiation of adipose-derived stromal vascular fractions through the *miR-152*/mesenchyme homeobox protein 2 (*MEOX2*) axis [130]. Several other bovine circRNAs, such as *circFUT10*, regulate PPAR-γ coactivator 1 beta (*PPARGC1B*) expression by competitive binding to *let-7c,* promoting adipocyte proliferation, inhibiting cell differentiation, and ultimately inhibiting bovine fat deposition [131]. Bovine *circADAMTS16* also inhibits adipocyte differentiation and promotes adipocyte proliferation by targeting *miR-10167-3p* [132]. *circSSBP2* inhibits the proliferation of bovine intramuscular preadipocytes by regulating the miR-2400/ N-myc downstream regulated 1 (*NDRG1*) axis [133]. *circHOMER1*, identified in pigs, plays a suppressive role in porcine fat deposition through *miR-23b* and SIRT1 [134]. *circMEF2C* modulates the proliferation and adipogenesis of porcine intramuscular preadipocytes through the miR-383/*671-3p*/MEF2C axis [135]. Another circRNA, *sus_circPAPPA2*, was found to inhibit fat deposition in castrated pigs through the *miR-2366*/glycerol kinase (*GK*) pathway [136]. In chickens, *circITGB5* has been reported to inhibit the proliferation and adipogenic differentiation of chicken intramuscular preadipocytes through the *miR-181b-5p*/*CPT1A* axis [137]. In sheep, Shen et al. discovered that circARID1A inhibits tail fat cell differentiation in Guangling large-tailed sheep through the *circARID1A*/*miR-493-3p*/*YTHDF2* axis [138]. Guo et al. found that *circRNA-5335* regulates the differentiation and proliferation of sheep preadipocytes via the *miR-125a-3p*/*STAT3* pathway [139].

**Figure 3:**
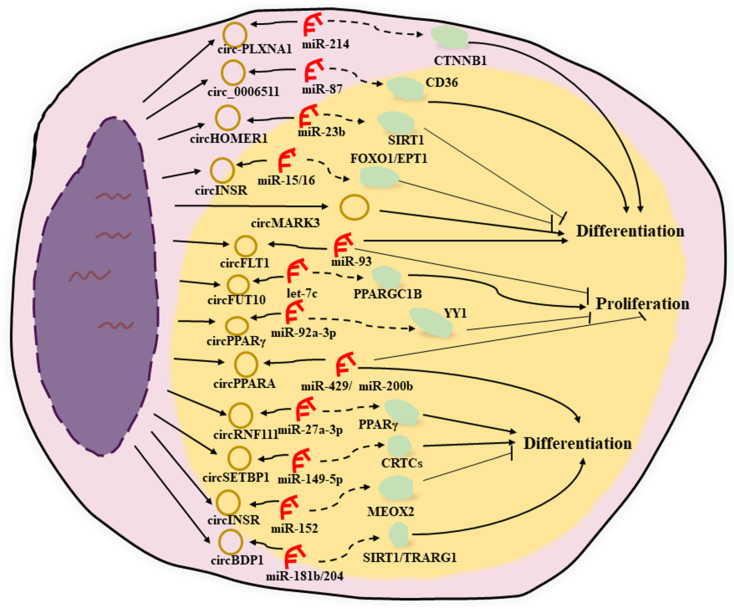
The regulatory roles and underlying mechanisms of circRNAs (shown as yellow circles) in fat deposition in farm animals. Short red segments and light green circles represent miRNAs and downstream genes, respectively. The dotted line and the solid line denote the regulation between circRNAs and their interacting genes.

Figure 3 illustrates the regulatory roles and underlying mechanisms of circRNAs in animal fat deposition. This section is summarized in Table 3. It is worth noting that *circINSR* can inhibit adipogenic differentiation in both cattle and sheep, indicating that some circRNAs also have conserved functions in different species.

## 4. Conclusions

Fat deposition has important impact on farm animal meat production and quality, which has prompted great interest in research on its formation and regulation. In addition to multiple transcription factors and regulatory genes, recent rapidly developing studies have demonstrated that ncRNAs play an essential role in fat deposition. Researchers have screened out a large number of ncRNAs involved in fat deposition in farm animals using systematic biological techniques, providing a theoretical basis and several regulatory targets for the breeding and improvement of farm animal meat quality. In this review, approximately 38 miRNAs, 21 lncRNAs, and 20 circRNAs were identified to participate in the fat deposition process of farm animals. Among these, miRNAs primarily regulate fat deposition by targeting the 3’ UTR regions of adipogenic genes to suppress their expression. In addition to a subset of lncRNAs that function by directly binding to proteins, the other lncRNAs and circRNAs mainly act through sponging miRNAs. This interaction prevents the miRNAs from binding to the 3’ UTR regions of their target genes, thus protecting the function of the target genes. This review provides valuable insights for future research regarding ncRNAs in fat deposition.

## 5. Outlook

With the continuous development of biological technologies and the improvement of ncRNA databases, researchers may expect to find additional ncRNAs involved in fat deposition in farm animals; however, the following scientific problems must be explored:

1. Research on ncRNAs in farm animals is relatively limited compared with medical studies, mainly because of the unavailability of comprehensive databases. In addition, the complexity of function and limited conservation of ncRNAs among species make them difficult to study. In recent years, the amount of high-throughput sequencing data on farm animals has risen. These data must be integrated into a comprehensive database.

2. Owing to the large size of some farm animals, the ncRNAs in the fat of cattle, sheep, and pigs have mainly been functionally verified in vitro at the cellular level, but there is a lack of in vivo validation studies. In the future, CRISPR/Cas9 gene editing technology, lentiviral vector construction, and other methods should be used to verify the functions of ncRNAs in animals in vivo.

3. In the medical field, ncRNAs are being used as biomarkers and therapeutic targets for a variety of diseases. However, few ncRNAs have been identified and developed as therapeutic targets for obesity-related diseases. Therefore, the discovery of the key ncRNAs involved in obesity-related diseases may contribute to the future treatment of such diseases.

4. ncRNAs perform biological functions via binding to DNA, RNA, and proteins; thus, we must identify the molecules that interact with ncRNAs to elucidate their molecular mechanisms of action. However, the approaches recently used to research ncRNAs related to fat deposition have been relatively simple and similar. We recommend that researchers make full use of the existing technologies, such as RNA pulldown, chromatin isolation by RNA purification (ChIRP), global RNA interaction with DNA by deep sequencing (GRID-seq), and RNA immunoprecipitation (RIP), to fully explore the regulatory network of ncRNAs in animal fat deposition.

## Figures and Tables

**Table 1:** miRNAs associated with fat deposition in farm animals.

Model	miRNA	Function	Direct Target(s)	Cell Culture Model
pig	miR-103	Pro-adipogenic	RAI14	preadipocytes
pig	miR-145	Anti-adipogenic	IRS1	dedifferentiated fat cells
pig	miR-181a	Pro-adipogenic	TNF-α	preadipocytes
pig	miR-199a-5p	Anti-adipogenic	CAV-1	preadipocytes
pig	miR-15a/b	Pro-adipogenic	FOXO1	preadipocytes
pig	miR-196a	Pro-adipogenic	--	preadipocytes
pig	miR-29b/29c	Anti-adipogenic	CTRP6	preadipocytes
pig	miR-125a-5p	Anti-adipogenic	KLF13	preadipocytes
pig	miR-127	Anti-adipogenic	HOXC6	preadipocytes
pig	miR-146a-5p	Anti-adipogenic	IR	preadipocytes
pig	miR-218-5p	Anti-adipogenic	ACSL1	preadipocytes
pig	miR-26a	Anti-adipogenic	ACADM, ACSL1	preadipocytes
pig	miR-29c	Anti-adipogenic	IGF1	bone marrow stromal cells
cattle	miR-143	Pro-adipogenic	--	preadipocytes
cattle	miR-1271	Pro-adipogenic	ATF3	preadipocytes
cattle	miR-381	Pro-adipogenic	KCTD15	preadipocytes
cattle	miR-130a/b	Anti-adipogenic	PPAR-γ, CYP2U1	preadipocytes
cattle	miR-107	Anti-adipogenic	APOC2	adipocytes
cattle	miR-330	Pro-adipogenic	SESN3	intramuscular preadipocytes
cattle	miR-484	Anti-adipogenic	MAP3K9	intramuscular adipocytes
cattle	miR-10167-3p	Anti-adipogenic	TCF7L1	preadipocytes
cattle	miR--365-3p	Anti-adipogenic	FKBP5	adipocytes
goat	miR-196a	Pro-adipogenic	MAP3K1	preadipocytes
sheep	miR-340-5p	Anti-adipogenic	ATF7	adipocytes
sheep	miR-27a	Anti-adipogenic	RXRα	preadipocytes
sheep	miR-124-3p	Anti-adipogenic	C/EBP-α	preadipocytes
sheep	miR-136	Anti-adipogenic	PPARGC1B	preadipocytes
sheep	miR-369-3p	Anti-adipogenic	--	preadipocytes
sheep	miR-33a	Anti-adipogenic	SIRT6	stromal vascular fraction cells
sheep	miR-432	Anti-adipogenic	DDI1	preadipocytes
chicken	miR-18b-3p	Anti-adipogenic	ACOT13	adipocytes
chicken	miR-122-5p	Pro-adipogenic	FABP5	preadipocytes
chicken	miR-106-5p	Anti-adipogenic	KLF15	preadipocytes
chicken	miR-128-3p	Anti-adipogenic	FDPS	intramuscular precursor adipocytes
chicken	miR-223	Anti-adipogenic	GPAM	preadipocytes
chicken	miR-125b-5p	Pro-adipogenic	ACSBG2	preadipocytes
chicken	miR-15a	Pro-adipogenic	ACAA1, ACOX1, SCP2	intramuscular preadipocytes
chicken	miR-140-5p	Pro-adipogenic	RXRG	intramuscular preadipocyte

**Table 2:** lncRNAs associated with fat deposition in farm animals.

Model	lncRNA	Function on Adipogenesis	Direct Target(s)	Cell Culture Model
pig	IMFlnc1	promote	miR-199a-5p	intramuscular preadipocyte
pig	lncIMF4	inhibit	--	intramuscular adipocytes
pig	lncMYOZ2	promote	MYOZ2	preadipocytes
pig	lncIMF2	promote	miR-217	intramuscular preadipocytes
pig	PU.1 AS lncRNA	promote	PU.1	preadipocytes
pig	PLA2G16-AS	--	PLA2G16	PK15
pig	LOC106505926	inhibit	FASN	preadipocytes
bovine	ADNCR	inhibit	miR-204	preadipocytes
bovine	BADLNCR1	inhibit	SREBP1/2	adipocytes
bovine	MIR221HG	inhibit	--	adipocytes
bovine	SERPINE1AS2	promote	PAI1	intramuscular adipocytes
yak	lncFAM200B	promote	--	preadipocytes
bovine	BNIP3	inhibit	--	intramuscular preadipocytes
bovine	BIANCR	promote	--	intramuscular adipocytes
buffalo	NDUFC2-AS lncRNA	promote	--	adipocytes
chicken	IMFNCR	promote	miR-128-3p/miR-27b-3p	intramuscular adipocytes
chicken	lncAD	promote	--	intramuscular preadipocytes
chicken	ZFP36L2-AS	promote	ACACA/PC	preadipocytes
chicken	lncEDCH1	inhibit	SERCA2	myoblasts
chicken	LNC6302	promote	--	abdominal preadipocyte

**Table 3:** circRNAs associated with fat deposition in farm animals.

Model	circRNA	Function on Adipogenesis	Direct Target(s)	Cell Culture Model
goat	circ_0006511	promote	miR-87	intramuscular preadipocytes
bovine	circBDP1	promote	miR-204/miR-181b	adipocytes
bovine	circPPARγ	promote	miR-92a-3p	primary adipocytes
bovine	circFLT1	promote	miR-93	adipocytes
bovine	circINSR	inhibit	miR-15/16	preadipocytes
bovine	circRNF111	promote	miR-27a-3p	preadipocytes
bovine	circFUT10	inhibit	let-7c	adipocytes
bovine	circADAMTS16	inhibit	miR-10167-3p	preadipocytes
bovine	circSSBP2	inhibit	miR-2400	intramuscular preadipocytes
buffalo	circMARK3	promote	--	buffalo adipocytes/3T3-L1
pig	circSETBP1	promote	miR-149-5p	intramuscular preadipocytes
pig	circPPARA	promote	miR-429/miR-200b	intramuscular preadipocytes
pig	circHOMER1	inhibit	miR-23b	preadipocytes
pig	circPAPPA2	inhibit	miR-2366	preadipocyte
pig	circMEF2C(2, 3)	inhibit	miR-383	intramuscular preadipocytes
sheep	circINSR	inhibit	miR-152	stromal vascular fractions
sheep	circARID1A	inhibit	miR-493-3p	adipocytes
sheep	circRNA-5335	promote	miR-125a-3p	adipocytes
duck	circ-PLXNA1	promote	miR-214	adipocytes
chicken	circITGB5	inhibit	miR-181b-5p	intramuscular preadipocytes

## Data Availability

Data sharing is not applicable to this article as no new data were created or analyzed in this study.
